# Frontiers of stem cell engineering for nanotechnology-mediated drug delivery systems

**DOI:** 10.5599/admet.2160

**Published:** 2024-02-14

**Authors:** Serap Yesilkır Baydar, Hatice Feyzan Ay, Rabia Cakir

**Affiliations:** 1 Faculty of Engineering and Architecture, Department of Biomedical Engineering, Istanbul Gelisim University, Avcilar, Istanbul, Türkiye; 2 Life Sciences and Biomedical Engineering Application and Research Centre, Istanbul Gelisim University, Avcilar, Istanbul, Türkiye; 3 Department of Bioengineering, Faculty of Chemistry and Metallurgical, Yildiz Technical University, Istanbul, Türkiye; 4 Biotechnology Institute, Health Institutes of Türkiye (TUSEB), Istanbul, Türkiye

**Keywords:** Stem cell nanotechnology, drug delivery technology, nanomaterials, stem cell-derived products

## Abstract

**Background and purpose:**

Cell biology approaches have gained a successful integration, development and application of nanotechnology with stem cell engineering and have led to the emergence of a new interdisciplinary field known as stem cell nanotechnology (SCN). Recent studies showed the potential and the advancement of developments for SCN applications in drug delivery systems. Cancer, neurodegenerative, muscle and blood diseases, cell and gene therapies, and tissue engineering and regenerative medicine applications are the important targets of SCN.

**Experimental approach:**

In this overview, we searched the literature using the common online websites for research and read the open access, full-text available articles since 2013.

**Key results:**

The studies vary according to the type of disease they targeted and the strategies they proposed, whether diagnostic or therapeutic. In addition to the use of stem cells, the utilisation of their membranes, secretomes, exosomes and extracellular vesicles with an appropriate nanotechnology strategy is also an aspect of the research.

**Conclusion:**

This brief overview of stem cell nanotechnology over the last ten years aims to provide insight into the frontiers of stem cell engineering for nanotechnology-mediated drug delivery systems.

## Introduction

Life on Earth began many years before the history of mankind and started to be discovered with the realization of mankind. It took tens of thousands of years for each element of life to be discovered and understood over time, and in the last two centuries, it has enabled incredible acceleration, enabling brand-new discoveries and inventions to be made. The successive discoveries made by life scientists and intra- and interdisciplinary studies have led to reshaping science and nature and the reorganization of the existing system for the purposes needed. One of the most exciting developments has been understanding how cytology's boundaries expand the deeper we go down. The re-engineering of the cell has led to incredible advances in agriculture, animal husbandry and the pharmaceutical industry, as well as many new approaches to medicine that can be used for diagnostic and therapeutic purposes. It would not be wrong to say that life scientists are also shaping the future with these exciting studies.

The perfect harmony of basic science and engineering applications, a portal has been opened from the macro world to the micro world [1]. With the discovery of a lot of space in the micro world, life scientists have started to rebuild today's medicine. Drug delivery systems, which deal with the transfer of many pharmaceutical products with healing properties to the targeted area of the body, are one of the important stakeholders of biomedicine. To date, very important developments have been achieved as next-generation therapeutics, including proteins, nucleic acids, monoclonal antibodies, living cells [2], and stem cells [3], which we have heard more about in the 2000s.

With the significant increase in the knowledge of cell scientists about stem cell biology in the last 25 years [4], as well as scientists focusing more on biotechnology studies and manufacturing products with unique properties of nanotechnology, an important field has arisen in the scientific community: stem cell nanotechnology [5-8]. In stem cell research, nanotechnology strategies have led to advanced capabilities attractive for an important emerging interdisciplinary field. With these advances, researchers need to find the fundamentals and limitations of the association between nanoparticles (NPs) and stem cells. If the dynamics of this relationship can be defined, it will also shed light on how future work on stem cell engineering as a drug delivery system via nanotechnology can be directed, which we will briefly focus on in this review [9]. Furthermore, this review also discusses key strategies in this niche research field, stem cell engineering for nano-based drug delivery systems, for the investigators of today’s biomedical applications.

## Review methodology

This report is a survey that concentrated on the literature in the English language and was carried out in databases such as ScienceDirect, PubMed and Google Scholar between 2013 and 2023 (except some book knowledge and leading studies from earlier years). The keywords for the search were chosen from the topics of cancer, neurodegenerative diseases, and muscle diseases and their applications in cell and gene therapies, tissue engineering, and regenerative medicine. Articles on stem cell engineering for nano-based drug delivery systems have been recognized by utilizing the search databases mentioned above.

## Drug delivery systems

Drug delivery systems are defined as technologies that help transport drugs into or throughout the body by ingestion, inhalation, absorption through the skin or injection, depending on the type of drug. An important goal of current drug delivery systems is to deliver the active substance to the appropriate site in the body without degradation and side effects, as these are the biggest challenges of the field. Improving existing systems, overcoming challenges and bringing new methods to a technological level where they can be used is an important aspect of drug delivery systems. Scientists continue to contribute significantly to our understanding of the physiological barriers to effective drug delivery and also contribute to the development of many new drug delivery methods emerging into clinical practice, such as in cancer, neurodegenerative and muscular diseases, etc. Unfortunately, treatments can sometimes have unacceptable side effects due to the interaction of medicines with healthy organs or tissues. Therefore, drug delivery systems continue to struggle to overcome these limitations. Drug delivery technologies have continued to advance since the first controlled-release formulation was introduced in 1950 [10-12]. Nowadays, the continuous development of nanotechnology has pioneered nano-drug delivery technology and has led to important applications in the field of drug delivery systems [1,13].

## Nanomaterials as drug delivers

Nanotechnology is one of the rapidly developing scientific fields of recent years and deals with nano-sized materials (particles, materials, *etc.*) [8,14-16]. The Scientific Committee on Emerging and Newly Identified Health Risks of the European Commission (SCENIHR) has defined natural or manufactured materials with at least 50 % of the size distribution between 1 and 100 nm as nanomaterials. Nanomaterials owe all their properties to their small volume, the atoms that make up their mass and the arrangement of these atoms in various shapes. When we look at nanobiomaterials, it is seen that the position of the constituent atoms on the surface of the material causes different biological, catalytic, electrical, steric, magnetic, mechanical and optical properties [17,18]. Due to these unique properties of nanomaterials, the budgets allocated to nanotechnology increase exponentially every year. Studies in this field have a huge market worldwide and are of great importance [19].

Traditional controlled release systems depend upon an established drug release rate regardless of environmental conditions during administration [13]. Fabrication of drug delivery carriers at the nanoscale usually depends on encapsulating chemotherapeutic drugs into organic and/or inorganic coats such as liposomes, polymeric micelles, biodegradable polymer nanoparticles and dendrimers. There are distinctive challenges to overcome for every delivery approach [20-22]. Nonspecific distribution, uncontrolled biodegradability and toxicological side effects are the most important disadvantages of conventional drug delivery systems. To overcome these challenges, nanoparticles have been used as drug delivery systems for diagnostic and therapeutic purposes, particularly to enhance the pharmacodynamic properties of drugs and to drive their penetration into tissues at the molecular level [23]. Biocompatible and non-toxic nanomaterials are currently reported to be synthesized by various methods, and of course, not only the method but also the types and properties of the nanomaterials make it possible for them to be an advantageous pharmacological product [24]. For these purposes, for a safer strategy, eco-friendly nanomaterial synthesis methods are started to be used for biomedical applications in order to eliminate the concerns of nanotoxicology. Green nanoparticles are seen to achieve an environmentally friendly approach by utilizing the active compounds of various biological entities such as bacteria, algae, cyanobacteria, yeast, actinomycetes, fungi and plants [25,26]. Preliminary *in vitro* studies report the promising potential of green nanoparticles for biological applications such as antioxidant, anti-inflammatory, antimicrobial, gene delivery and drug development to fight chronic diseases [26-35], but stem cell-based therapies are rare.

With existing knowledge and principles, the fabrication of multifunctional nanomaterials with a convenient size and homogeneous structures is still the focus of the field. However, the organic or inorganic partners that the synthesised nanoparticles will be used together as a drug transport system and the type of disease to be targeted also require very deep and meticulous investigations.

With the understanding that nanomaterials can penetrate the cell membrane and enter the cell thanks to their size and surface properties, current approaches have emerged in recent years.

## Stem cell nanotechnology

Within the body are stem cells that can ensure the continuity of the living tissues and organs in which they are and have the capacity to proliferate and differentiate themselves. Stem cells with various differentiation potentials, such as totipotent, multipotent, and unipotent, can develop into diverse types of cells, including liver cells, nerve cells, and cardiomyocytes according to the physiological needs of the body, depending on which tissue/organ needs regeneration [10,36-38]. Stem cells can be obtained from a variety of sources, such as peripheral blood, cord blood, bone marrow, adipose tissue, and dental pulp, for scientific research and/or medical purposes [36,39-41].

Today, we know that nanotechnology is expanding into the isolation, differentiation, and imaging of stem cells and the engineering for biomedicine. However, as in all interdisciplinary fields, stem cell nanotechnology encounters several challenges. It is necessary to understand better the process of interaction between nanomaterials and stem cells since it is difficult for the act of metabolizing nanomaterials modified to improve the stem cells' functions [9]. While the therapeutic use of stem cells as a part of the drug delivery system is important, the state-of-the-art technology to precisely control the engineering of stem cell behaviours with nanomaterials is still under development [42]. Nevertheless, it is encouraging that improvements in this field will enable drug delivery systems to be designed more effectively.

## Stem cell nanotechnology applications as drug delivery systems

**Figure 1.**
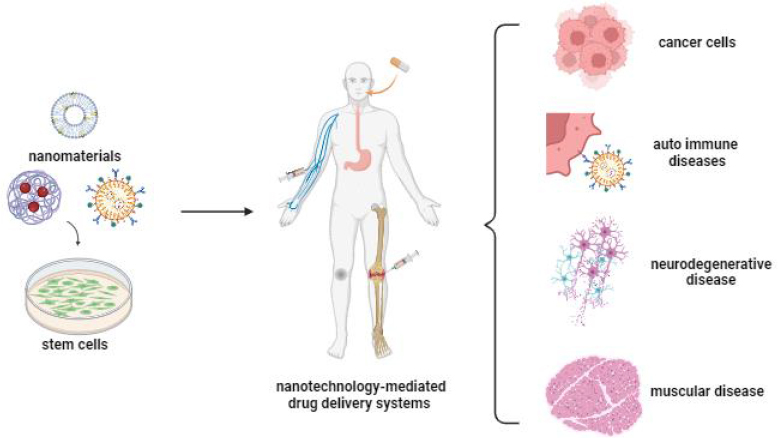
Illustration of drug delivery systems via nanotechnology-mediated stem cell engineering (created with BioRender.com)

In recent years, with the realisation that stem cells can accompany nanoparticles with superior properties, fascinating scenarios have started to emerge regarding the potential use of these cells in the treatment of various types of diseases [9]. As an important tool for cellular therapies, stem cells offer an important and new field of medical applications for the diagnosis and therapy of various disorders such as blood diseases, tissue regeneration, tissue repair, liver failure, cancer, nervous system damage, autoimmune diseases, *etc.* (Figure 1) [43-45].

In addition to stem cells’ low immunogenicity and high targeting ability, they produce paracrine factors during the repair process, indicating that stem cells have the appropriate capacity for drug delivery systems. The paracrine factors have antiapoptotic, proangiogenic, and accelerated cell proliferation effects, which are essential to support the tissue [10,46]. Furthermore, they may show an inherent affinity for damaged tissues or tumour sites, and their targeting ability can be enhanced by gene and nanotechnology applications, thus enabling specific targeting and delivery of genes and drugs for various organs [24]. Stem cells and stem cell membranes can be used directly in the drug delivery system, and extracellular vesicles and exosomes derived from stem cells are also of big interest because of their small size, high drug-loading efficiency and long half-life [3]. Furthermore, stem cells and their secretomes have been utilized to enhance the efficiency of NPs in drug delivery. There are studies reporting that coating NPs with stem cells or forming complexes can enhance the targeting ability and therapeutic effect of NPs. Fan *et al.* [47] reported that coating NPs with stem cells or forming the complexes can increase the targeting ability and therapeutic effect of NPs.

In this review, we briefly addressed serious chronic illnesses and stem cell engineering applications as drug delivery systems via nanotechnology for these diseases.

### Cancer treatments

Today, nanoparticles are used to eliminate the systemic toxic effects of drugs used in cancer treatment, to ensure and facilitate effective drug targeting, and to increase cellular uptake efficiency, anti-proliferative effect, and anti-tumour activity [48].

The effects of different nanostructures modified with stem cell membranes loaded with cancer drugs on cancer diseases have been examined in various studies. Pascucci *et al.* [49] investigated the targeting and anti-tumour effects of mesenchymal stromal cells (MSCs) and membrane microvesicles (MVs) secreted by MSCs, with regard to the ability to uptake and release the drugs of MSCs. In the study, the mouse SR4987 line that was used as the MSC was loaded with paclitaxel (PTX; SR4987PTX and SR4987PTX-MVs)) and SR4987PTX was found to secrete substantial amounts of PTX. In addition, SR4987PTX and SR4987PTX-MVs were reported to exhibit a potent anti-proliferative effect and anti-tumour activity on human pancreatic adenocarcinoma CFPAC-1 Liu *et al.* [50] investigated the effects of doxorubicin (DOX) loaded superparamagnetic iron oxide nanoparticles (SPIO NPs) coated with mesenchymal stem cell (MSC) membranes on colon cancer. DOX-SPIO NPs were coated with MSC membranes (DOX-SPIO@MSCs) and DOX-SPIO NPs and DOX-SPIO@MSCs were compared. The *in vitro* studies using MC38 cells proved that the DOX-SPIO@MSCs increase the efficiency of cellular uptake, effects of apoptosis and anti-tumour and reduce the response of the immune system. In MC38 tumour-bearing C57BL/6 mice experiments, it has been shown that DOX-SPIO@MSCs increase the effectiveness of tumour treatment while reducing systemic side effects. In another study using SPIO NPs, the new stem cell membrane (STM) was used. In the study, Lai *et al.* [51] reported STM-camouflaged SPIO NPs. Macrophage uptake of STM-SPIO NPs was found to be less than that of SPIO NPs. Moreover, under the application of an alternating magnetic field (AMF), STM-SPIO NPs have been shown to successfully induce cancer cell death. In another study, stem cell membrane-coated gelatine nanogels (SCMGs) were developed for high tumour targeting, and DOX-loaded stem cell-coated gelatine nanogels (SCMGs-DOX) were formed by encapsulating DOX into these nanogels. Compared with gelatinDOX and free-DOX, SCMGs-DOX has been shown to have higher anti-tumour therapeutic efficacy and SCMGs slow down tumour growth. Moreover, it has been shown that there are no important side effects in the tissues of mice due to its tumour-targeting ability [52]. Suryaprakash *et al.* [53] also focused on MSC spheroids and developed a hybrid spheroid/nanomedicine system containing a drug-loaded nanocomposite that entrapped TRAIL-expressing MSC spheroids for treatments of glioblastoma. In this study, while this system provides an active drug delivery with specific targeting, MSC has been shown to increase tumour tropism, exhibit higher nanocomposite retention in tumour tissue, and increase the amount delivered to the tumour site.

Recent studies focus on exosomes and extracellular vesicles (EVs). Bagheri *et al.* [54] used exosomes derived from mouse MSCs for the treatment of colorectal cancer and loaded DOX into exosomes tagged with 5TR1 aptamer. The 5TR1 aptamer-decorated exosomes carrying DOX (DOX@exosome-apt) have demonstrated that they can significantly affect tropism and inhibition in MUC1-positive cancer cells *in vitro*. Moreover, *in vivo* studies have demonstrated that DOX@exosome-apt can significantly inhibit tumour growth. In another study, the encapsulation of DOX in these desialylated MSC-derived EVs after sialic acids on the surface of MSC-derived EVs were removed by neuraminidase. The results showed that for targeting hepatocellular carcinoma, DOX-loaded desialylated EVs increased the cellular uptake and targeting efficiency and had better inhibition both *in vitro* and *in vivo* [55].

### Neurodegenerative disorders

One of the popular therapeutic areas of stem cells is for neurodegenerative diseases (NDs). In the treatment of NDs, especially mesenchymal and neural stem cells are frequently used as stem cell types.

The protective effect of 6-hydroxydopamine (6-OHDA)-induced neurotoxicity of extracellular vesicles (EVs) isolated from human fibroblast and neural stem cells was investigated. Both EVs were able to reduce ROS and apoptotic pathways. In addition to that, EVs isolated from neural stem cells were reported to reduce dopaminergic neuron loss and induce downregulation of pro-inflammatory factors [56]. Another study investigated the effect of EVs secreted by adipose-derived MSCs (hADSC) against encephalomyelitis-induced neurodegeneration. hADSC and hADSC-EV caused a significant reduction in the inflammation score and demyelination areas. Furthermore, hADSC-EVs were discovered to attenuate induced encephalomyelitis by reducing leukocyte infiltration and demyelination [57]. In another 6-OHDA-induced model, it has been reported that dextran-coated iron oxide nanoparticles (Dex-IO NPs)-labeled hMSCs can increase therapeutic effectiveness, promote their migration to damaged neuron sites, and stimulate their differentiation [58]. In a study with exosomes, the effect of exosomes secreted from MSCs in the therapy of multiple sclerosis has been evaluated and it has been reported that exosomes stimulated by IFNγ (IFNγ-Exo) reduce demyelination and neuro-inflammation and increase the regulatory T cells in spinal cords [59].

The role of stem cells is not only to treat or prevent neural damage such as neuro-inflammation, neurodegeneration, and demyelination but also to provide an excellent strategy for the proliferation, differentiation, and regeneration of neurons. The neural stem cells (NSCs) and carbon nanotubes (CNTs) were used together in a study to promote neural regeneration. In the study, CNT/NSCs provided an increasing propensity to differentiate into neurons. It has also been reported to repair cognitive deficits and neurodegenerative changes in trimethyltin-induced neurodegeneration [60]. For carbon nanotechnology, the effect of fullerene carbon 60 (C60) on neuronal differentiation was also investigated. In the research, alanine-bearing C60 (Ala-C60) was synthesized and it was reported that Ala-C60 could promote the proliferation and differentiation of NSCs. Importantly, Ala-C60 showed an antioxidative effect by increasing cell viability in NSCs treated with hydrogen peroxide [61].

### Muscular diseases

Stem cell research for the treatment of muscular diseases is also gaining interest. Magnetic systems are used to ensure that stem cells are retained in the skeletal muscle for a long time; thus, both effective targeting and long-term retention can be achieved. Magnetic targeting can be provided with magnetic agents such as SPIONs or by creating an external magnetic field [62]. In a study by Kono *et al.* [62], they prepared magnetized MSCs using magnetic anionic liposome/atelocollagen complexes and investigated their retention efficiency and immunomodulatory effects for the sarcopenia treatment. The results showed that magnetized MSCs had a higher retention in skeletal muscles. It was also discovered that IL-6 and IL-10 were upregulated, while TNF- and IL-1 were downregulated in inflamed skeletal muscle. Zhang *et al.* [63] developed MSC secretome-loaded PLGA NPs (MSC-Sec NPs) for the treatment of osteoporosis. They then coated these NPs with human microvascular endothelial cells (HMECs) membranes expressing C-X-C chemokine receptor type 4 (CXCR4) to produce MSC-Sec/CXCR4 NPs. The results showed that the stem cell secretome, like real stem cells, contained osteoprotegerin and BMP-2 and exhibited their long-term releases. Moreover, while CXCR4 increased MSC-Sec/CXCR4 NP accumulation in bone, MSC-Sec/CXCR4 NPs were discovered to be successful in promoting osteogenesis, inhibiting osteoclast differentiation, and reducing ovariectomy-induced bone mass attenuation.

### Cell and gene therapies

The goal of gene therapy is to replace disease-causing inherited mutations and an abnormal or dysfunctional gene with a healthy functional form [37,64]. Gene therapy involves the use of exogenous nucleic acids such as genes, gene segments, oligonucleotides, miRNAs or siRNAs. The use of nanoparticles as carriers in gene therapy is notable for their targeting ability, variety of functionalization, and low immunogenicity and toxicity [65].

For an effective siRNA therapy, siRNA-loaded iron oxide (Fe3O4) NPs were first coated with polydopamine (PDA) (Fe3O4@PDA) and then coated with MSCs to generate a membrane. The Fe3O4@PDA−siRNA@MSCs, which retained photothermal ability, showed excellent tumor targeting ability, effectively delivered siRNA to DU145 cells and induced silencing of the endogenous Plk1 gene. The results proved that the siRNA@MSCs complex had enhanced antitumor activity [66]. Shi *et al.* [67] developed a CD117 antibody-modified lipid nanoparticles (LNP) capable of delivering siRNA and mRNA to hematopoietic stem and progenitor cells (HSPCs) *in vivo* for hematopoietic stem cell gene therapy. It revealed that efficient gene regulation was achieved in HSPCs and that the stemness and functionality of transfected HSPCs were preserved.

### Tissue engineering

Stem cell-based studies for tissue regeneration and cell differentiation have also become interesting recently. Ren *et al.* [68] used nanoscale zeolitic imidazolate framework-8 (ZIF-8) modified to direct MSCs into a lineage of osteoblasts for the purpose of regulating the differentiation of MSCs during tissue regeneration. ZIF-8 NPs were encapsulated using stem cell membranes (SCMs) to mimic natural molecules, and thus, SCM/ZIF-8 NPs increased their specific internalization toward MSCs and the osteogenic capability of MSCs. Also, *in vivo* SCM/ZIF-8 NPs have been shown to promote bone tissue formation in the femoral bone. In a study to stimulate neural differentiation of MSCs, MSCs cultivated on the reduced graphene oxide membrane (rGO-M) were stimulated by a rotating magnetic field (RMF). *In vitro* experiments indicated that MSCs on rGO-M induced by RMF were able to express neuron-specific genes and proteins; *in vivo* experiments exhibited that exogenous MSCs on rGO-M can differentiate into neural cells driven by RMF [69].

Choe *et al.* [70] developed bioinks using bone morphogenetic protein-2 (BMP-2) encapsulated NPs to bioprint MSC-loaded scaffolds for bone tissue engineering and used alginate and poly(lactic-co-glycolic acid (PLGA) nanoparticles. It has been shown that the combined use of PLGA and alginate improves the mechanical properties and printability of bioink. The developed bioink exhibited excellent cell viability and osteogenesis while increasing calcium deposition and alkaline phosphatase activity.

### Regenerative medicine

One of the most active areas in the global biomedical area since the turn of the 20^th^ century have been stem cell and regenerative medicine technologies, playing an indispensable role in protecting human life and health and improving human survival quality [10,39]. In the framework of regenerative medicine applications, exosomes derived from stem cells, similar to stem cells, play different roles in various phases of wound healing. Exosomes can stimulate cell proliferation and neovascularization by modulating the inflammatory response [71].

One study evaluated synthetic analogs of MSCs for acute myocardial infarction treatment. In the study, the secreted factors from MSC were packaged into PLGA microparticles and then coated with MSC membranes to form Synthetic MSC (SynMSC). *In vitro* and *in vivo* studies showed that SynMSC promoted cardiomyocyte functions and angiogenesis, protective effects on heart morphology, and mitigated left ventricular remodeling [72]. Lee *et al.* [73] combined iron oxide nanoparticles (IONPs) with MSC and MSC-derived extracellular nanovesicles (NVs) for the treatment of myocardial infarction. IONP-NVs have been found to increase retention in infarcted myocardium, reduce apoptosis and fibrosis, and improve angiogenesis and cardiac function. Furthermore, they displayed antifibrotic, antiapoptotic, anti-inflammatory, and proangiogenic effects on cardiac therapeutic mechanisms.

MSC and nanoparticle complex have also been used for the treatment of rheumatoid arthritis (RA) and developed a drug delivery system of MSC membrane-encapsulated PLGA. Gu *et al.* [74] coated the adipose-derived MSC (ADSC) membrane on PLGA and then loaded with tacrolimus (FK506). This newly developed nanosystem was able to inhibit inflammation by enhancing the anti-inflammatory effect of tacrolimus and also enhancing the expression of anti-inflammatory factors while decreasing the expression of pro-inflammatory factors.

## Conclusions

Drug delivery systems based on nanotechnology are a speculative field of interest in many countries, and their importance in biology, biotechnology, chemistry, medicine, physical sciences, and genetic engineering is predicted to grow in the future. In recent years, with a better understanding that *there is still plenty of room at the bottom* [75], research into the interactions between stem cell nanotechnology and nanostructures has become a novel and interesting field. The potential of nanotechnology to main advances in drug delivery for diseases such as cancer, neurodegeneration, and so on has been extensively speculated. Specifically, the effect of nanoparticles combined with stem cells for targeting represents a new interdisciplinary frontier in drug delivery. In drug delivery, various nanomaterials have been used for transferring, transporting, and molecular imaging drugs or active substances; designed nanostructures have been used in stem-cell engineering. The multidisciplinary approaches of nanotechnologies for discovering novel systems and modifying those could have enormous potential to benefit human health. In the review we prepared, the studies using stem cells and nanoparticles have been shown to have more effective results compared to strategies using stem cells or nanoparticles alone. Therefore, it is possible to imagine a future in which new treatment strategies will be created by participating in the treatment of diseases by targeted structures.

## Future aspects

The innovative development and use of stem cell engineering and nanotechnology provide a global standpoint on research and combine the spin-off effects and advantages for human disorders in general. Traditional sciences such as physics, chemistry, biology, and materials science help to bring together the necessary knowledge and skills to create these innovative nanotechnologies. Recent advances in the field of nanotechnology have led to the revolution in biomedicine aimed at improving the life quality of humans, and novel approaches have enabled rapid and effective solutions for the treatment of diseases. With advancements in biomedicine, nanomedicine advances have also been made possible. Incorporating stem cell-nanostructure-nanodrug interactions and developing methods to create nanoscale surface features has become a significant clinical target of nanomedicine. It is obvious that this results in novel and more effective strategies when stem cell nanotechnology strategies are used to prevent and treat many diseases. In addition to studies on diseases, its potential advantages in biomedical applications, tissue engineering, and regenerative medicine are demonstrated. This makes it clear that nanotechnology will play a crucial role in the search to find a solution for future medical problems. However, nanotechnology, along with its opportunities, can also bring challenges, such as the long-term effects of nanostructures on the body, the safety of nanoformulations, and possible nanotoxic effects. Researchers focus on better strategies to overcome these critical challenges and apply nanotechnology to stem cell research.
